# MPINet: Metabolite Pathway Identification via Coupling of Global Metabolite Network Structure and Metabolomic Profile

**DOI:** 10.1155/2014/325697

**Published:** 2014-06-25

**Authors:** Feng Li, Yanjun Xu, Desi Shang, Haixiu Yang, Wei Liu, Junwei Han, Zeguo Sun, Qianlan Yao, Chunlong Zhang, Jiquan Ma, Fei Su, Li Feng, Xinrui Shi, Yunpeng Zhang, Jing Li, Qi Gu, Xia Li, Chunquan Li

**Affiliations:** ^1^College of Bioinformatics Science and Technology, Harbin Medical University, Harbin 150081, China; ^2^Department of Mathematics, Heilongjiang Institute of Technology, Harbin 150050, China; ^3^Department of Computer Science and Technology, Heilongjiang University, Harbin 150080, China; ^4^Department of Medical Informatics, Harbin Medical University, Daqing Campus, Daqing 163319, China

## Abstract

High-throughput metabolomics technology, such as gas chromatography mass spectrometry, allows the analysis of hundreds of metabolites. Understanding that these metabolites dominate the study condition from biological pathway perspective is still a significant challenge. Pathway identification is an invaluable aid to address this issue and, thus, is urgently needed. In this study, we developed a network-based metabolite pathway identification method, MPINet, which considers the global importance of metabolites and the unique character of metabolomic profile. Through integrating the global metabolite functional network structure and the character of metabolomic profile, MPINet provides a more accurate metabolomic pathway analysis. This integrative strategy simultaneously captures the global nonequivalence of metabolites in a pathway and the bias from metabolomic experimental technology. We then applied MPINet to four different types of metabolite datasets. In the analysis of metastatic prostate cancer dataset, we demonstrated the effectiveness of MPINet. With the analysis of the two type 2 diabetes datasets, we show that MPINet has the potentiality for identifying novel pathways related with disease and is reliable for analyzing metabolomic data. Finally, we extensively applied MPINet to identify drug sensitivity related pathways. These results suggest MPINet's effectiveness and reliability for analyzing metabolomic data across multiple different application fields.

## 1. Introduction

The development of high-throughput metabolomics technology, such as nuclear magnetic resonance and approaches based on mass chromatography, has enabled us to obtain metabolomic profiles of large numbers of metabolites [1]. The increasing availability of high-throughput data and large-scale high-quality pathway sources, such as KEGG [2] and Reactome [3], provides the potential for understanding these metabolomic data at the pathway level. Many currently available pathway-identification approaches are effective in transcriptomics [4, 5], though a method that effectively incorporates both global biological network structure and metabolomic profile is urgently needed.

Several computational approaches for metabolite pathway analysis have been developed recently, including overrepresentation analysis (ORA) and set enrichment analysis (SEA) [6]. ORA is widely used by researchers from a statistical perspective. It subjects a list of interesting metabolites (e.g., differential metabolites) to statistical analysis to detect whether the given metabolite set is overrepresented in a predefined pathway. For example, metabolite biological role (MBRole) [7] and metabolite pathway enrichment analysis (MPEA) [8] used ORA to perform pathway analysis based on metabolite sets. Metabolite set enrichment analysis (MSEA) [9] is a classic method of SEA, which involves enrichment analysis based on the whole list of metabolites identified in the profile, and it is also taking into consideration metabolite concentrations. There is no doubt that pathway analysis should involve a statistical model in order to reduce the incidence of false positive identification of differential metabolites based on a computational approach. However, both ORA and SEA consider a pathway as a simple metabolite set; they ignore functional interactions among metabolites and treat all metabolites equivalently. From a biological view, some metabolites should receive more attention than others and this is defined as the nonequivalent roles of metabolites in the pathway. Recently, a topology-based pathway analysis method, metabolomics pathway analysis (MetPA), which considers the local nonequivalence of metabolites in an individual pathway, was designed by Xia and Wishart to effectively improve pathway identification [10]. However, individual pathways are components of biological networks, and metabolites in different pathways also have functional interactions [11, 12]. A network-based approach that takes account of functional interactions to evaluate the nonequivalence of metabolites from a global perspective is therefore more suitable.

Also, the currently available approaches of metabolomic functional analysis ignore several common important aspects. Firstly, from a biological point of view, some metabolites participate in many pathways; they are referred to as common metabolites, while other metabolites only participate in a few pathways, which are referred to as pathway-specific metabolites. If a pathway identification method is mainly based on common metabolites, there will be more false positive pathways due to the presence of metabolites that are involved in multiple pathways. In contrast, it is more reliable that metabolites within an identified pathway tend to be pathway-specific as these dysfunctional metabolites only particularly involved in this pathway. Thus, these pathway-specific metabolites are more important in the pathway identification.

Secondly, dysfunction of metabolites may be compensated for by their functional partners in the biological network. Metabolites involved in a number of pathways usually have many functional partners, while other metabolites only participate in a few pathways and thus have few functional-compensation partners. If metabolites within a pathway tend to be pathway-specific, its deregulated signals are likely to be amplified to the entire pathway. In contrast, if the metabolites in a pathway tend to have many functional partners in the biological network, the dysregulation signal is more likely to be alleviated. Thus, the nonequivalence roles of metabolites should be considered in order to identify pathways correctly.

Finally, from the perspective of metabolomic technology, there is bias existing in metabolite identification. Most current metabolomic technologies usually only analyze a small fraction of the entire metabolome (5–10%) [6], and the identified metabolites are not randomly selected. These identified metabolites in the profiles are preferred to be well studied. This may be due to the fact that metabolite identification depends heavily on* a priori* knowledge [13, 14]. This metabolite identification bias will lead to the subsequent pathway identification bias because these well-studied metabolites usually reside in fundamental pathways, such as glutathione metabolism, or the citrate cycle. These fundamental pathways are thus likely to be inappropriately identified when metabolites are treated equivalently in pathways. In contrast, pathways that do not contain many well-studied metabolites (i.e., metabolites that are difficult to be identified in the profile) are more likely to be ignored by most of the current methods. It is therefore essential to consider the bias from metabolomic experimental technology. Furthermore, in contrast to transcriptome analysis, which can provide thousands of differential molecules for functional analysis, metabolomics usually use only dozens of metabolites for pathway identification. However, most of the metabolite pathway analysis methods currently proposed were originally designed for transcriptomics.

In this study, we developed a network-based pathway-analysis method called MPINet that considers both the global nonequivalence of metabolites and the bias from metabolomics experimental technology. In addition, a classical statistical model is also integrated. We constructed a human metabolite functional network. The global nonequivalence of metabolites within pathways refers to the different importance of metabolites and was evaluated based on the global functional interactions of metabolites within networks. Initial bias scores of metabolites were assigned based on the metabolomic profile, and a monotonic cubic regression spline model was then fitted to integrate the global nonequivalence scores and the initial bias scores of metabolites, and weights were assigned to the metabolites (nodes) in the network. Finally, the pathway weight, which was calculated based on the global node-weighted network, was used as the parameter for Wallenius approximation [15] to evaluate the significance of the pathway. We applied MPINet to four datasets and demonstrated the ability of MPINet to identify biologically meaningful pathways successfully and reproducibly across multiple different application fields. MPINet has been implemented as a free web-based (http://bioinfo.hrbmu.edu.cn/MPINet/) and R-based tool (http://cran.r-project.org/web/packages/MPINet/), supporting 3350 human pathways across 10 databases.

## 2. Materials and Methods

### 2.1. Datasets and Processing

We analyzed one metastatic prostate cancer dataset, two type 2 diabetes datasets, and one drug-sensitivity dataset. These datasets were obtained from metabolomics experiments or manually extracted from the literature.

#### 2.1.1. Metastatic Prostate Cancer Dataset

The metabolomic profile of prostate cancer was obtained from the study of Sreekumar et al. [16]. It included 16 tissue samples from benign adjacent prostate, 12 from localized prostate cancer, and 14 from metastatic prostate cancer samples [16]. In this study, we used the localized prostate cancer and metastatic prostate cancer samples as a case-control study. Differential metabolites were determined by Wilcoxon's rank-sum test (*P* < 0.1). Finally, 92 metabolites were identified as metastatic prostate cancer differential metabolites and used for pathway analysis.

#### 2.1.2. Type 2 Diabetes Datasets

We analyzed two type 2 diabetes datasets: type 2 diabetes dataset 1 and type 2 diabetes dataset 2. We extracted type 2 diabetes-associated metabolites from the HMDB database [17] and text mining (supplementary Table S1 in Supplementary Material available online at http://dx.doi.org/10.1155/2014/325697). Finally, 65 metabolites were identified as type 2 diabetes-associated metabolites.

Dataset 2 was obtained from Suhre et al. [18]. Multiplatform metabolomic profiles, including 482 metabolites, were detected from 40 individuals with type 2 diabetes and 60 control individuals. We selected the differential metabolites from the data preprocessed by Suhre et al. (*P* < 0.05) and converted the metabolite names to PubChem CIDs. Sixty-six metabolites related to type 2 diabetes were used for subsequent analysis.

#### 2.1.3. Drug-Sensitivity Dataset

A total of 121 drugs selected from Weinstein et al. [19] were analyzed. Drug-sensitivity data based on GI50 values were obtained from the CellMiner database [20], and metabolite measurement data were obtained from Metabolon, downloaded from http://dtp.nci.nih.gov/mtargets/download.html. The drug-sensitivity dataset included the −log(GI50) data for drugs in replicated experiment across NCI-60 cell lines. For each drug, the −log(GI50) values from different experiments were averaged. The metabolite measurement dataset included 352 metabolites across 58 cell lines, of which 160 named metabolites mapped to 159 distinct PubChem CIDs.

For each drug, we calculated the Pearson correlation of the drug −log(GI50) value and the metabolite measurements obtained in the experiment by Metabolon across 58 NCI-60 cell lines. The Benjamin method was used to correct the *P* value. The significant drug-sensitivity-related metabolite cutoff was set at 60%. MPINet was then applied to drug-sensitivity-related metabolites to identify drug-sensitivity-associated pathways.

### 2.2. Methods

**Figure 1:**
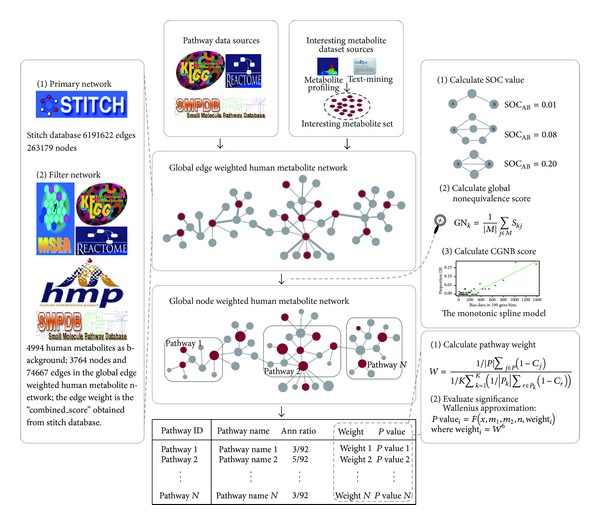
Schematic overview of MPINet.

We have implemented MPINet as a freely available R-based (http://cran.r-project.org/web/packages/MPINet/) and web-based tool (http://bioinfo.hrbmu.edu.cn/MPINet/), supporting 3350 human pathways across 10 databases, including KEGG, Reactome, PID, and Wikipathways from ConsensusPathDB [21]. Input only requires a list of the metabolites of interest (e.g., differential metabolites).  Figure 1 gives a schematic overview of MPINet.

#### 2.2.1. Construction of the Global Edge-Weighted Human Metabolite Network

The preliminary metabolite interaction network was downloaded from the STITCH database (http://stitch.embl.de/) [22]. We constructed the global edge-weighted human metabolite network from the preliminary network as follows. First, we extracted stereospecific compound interactions from the chemical-chemical link file and used the “combined score” in STITCH, as the initial edge weight. Second, we collected human metabolites from a wide range of databases, including KEGG [2], HMDB [17], Reactome [3], MSEA [9], and SMPDB [23]. We obtained the total of 4994 human metabolites from these five databases. Third, we extracted human metabolite interactions from the preliminary network obtained in the first step. We extracted the interacting pair if both of the metabolites in the interacting pair were included in the 4994 human metabolites. Finally, a global edge-weighted human metabolite network was constructed, which contained 3764 nodes and 74667 weighted edges (Table S2).

#### 2.2.2. Calculating the Global Nonequivalence Scores of Metabolites Based on the Network

Based on the notion that dysfunction of metabolites with strong functional-interaction partners will be more easily compensated for, we defined a global nonequivalence score (GN score) to measure the functional interaction between a metabolite and its functional partners in the global metabolite network.

Firstly, we quantified the functional interactions between each pair of metabolites in the network by calculating the global connection strength (GCS) for each metabolite pair. The GCS measure was defined as the modified version of the strength of connection (SOC) measure in Campbell et al. [24], which measured the connection strength between metabolites from a global perspective by considering both the number and length of multiple paths between two metabolites in the network. A detailed description of how the GCS values were calculated was included in the supplementary text. Higher GCS values indicate stronger functional interactions between the metabolite pairs.

We then defined the GN score of a given metabolite as the mean of the GCS values between it and the other metabolites in the network. A high GN score indicates that the metabolite has strong functional interactions with its functional partners, and dysfunction of these metabolites is thus likely to be compensated for. In contrast, metabolites with low GN scores have weaker interactions, and their dysfunction is less likely to be compensated for, which suggests that metabolites should be paid more attention.

#### 2.2.3. Calculating the Combined Global Nonequivalence and Bias Scores of Metabolites

Our goal was to consider simultaneously the global nonequivalence of metabolites in a pathway and the bias in pathway identification based on metabolomic data. These two critical factors are not independent, given that metabolites with a high GN score are more likely to be identified in the profile and to be identified as differential. A simple sum of the values for these two factors is thus not adequate. We therefore used a monotonic cubic regression spline model [25] with six knots and a monotonicity constraint to integrate the GN scores and the initial bias scores. This monotonic model quantified the probability of metabolites being differential and compensated for as a function of GN score.

The cubic regression spline with *k* knots can be represented as

(1) $$\begin{array}{c} y\left(x\right)=\beta_{1}b_{1}\left(x\right)+\beta_{2}b_{2}\left(x\right)+\cdots +\beta_{k}b_{k}\left(x\right), \end{array}$$

where *b*_*i*_(*x*) is the basis cubic function at knot *i*,  *i* = 1 ⋯ *k*. For a given response *y*_*j*_ and covariates *x*_*j*_,

(2) $$\begin{array}{c} y_{j}=y\left(x_{j}\right)=\beta_{1}b_{1}\left(x_{j}\right)+\beta_{2}b_{2}\left(x_{j}\right)+\cdots +\beta_{k}b_{k}\left(x_{j}\right), \end{array}$$

where the *y*_*j*_ in this study is a binary value for the corresponding metabolite, 1 for differential metabolites and 0 for nondifferential metabolites in the network; *x*_*j*_ is the GN score for the corresponding metabolite. Suppose that the *k* knots are *x*_1_*, *x*_2_*, …, *x*_k_*, which are placed at the quantiles of the distribution ofthe GN score vector; then the basis cubic function *b*_*i*_(*x*) can be represented as

(3) $$\begin{array}{c} b_{i}\left(x\right)=\varphi \left(x,x_{i}^{*}\right). \end{array}$$

Supposing that there are *M* metabolites in the network, the binary initial bias score vector of these *M* metabolites *Y* can be represented as

(4) $$\begin{array}{c} Y=\left[\begin{array}{c} y_{1}\\ y_{2}\\ \vdots \\ y_{M} \end{array}\right]. \end{array}$$

The cubic spline model can then also be represented as

(5) [y1y2⋮yM]=[φ(x1,x1∗),φ(x1,x2∗),…,φ(x1,xk∗)φ(x2,x1∗),φ(x2,x2∗),…,φ(x2,xk∗)⋮φ(xM,x1∗),φ(xM,x2∗),…,φ(xM,xk∗)][β1β2⋮βk] +[ε1ε2⋮εM],

where *y*_*j*_, *x*_*j*_  (*j* = 1 ⋯ *M*) are the initial bias score and the GN score of the corresponding metabolite, respectively. Consider *y*_*j*_ = 1 for an interesting metabolite; otherwise *y*_*j*_ = 0. The cubic spline model can be simplified as

(6) $$\begin{array}{c} Y=X\beta +\varepsilon , \end{array}$$

where *X* is the model matrix, *β* is the parameter vector that contains *β*_1_, *β*_2_,…, *β*_*k*_. The penalized constrained least squares with monotonicity constraint are used to evaluate the parameter vector *β*, to minimize

(7) $$\begin{array}{c} {||Y-X\beta ||}^{2}+\lambda \beta^{T}S\beta . \end{array}$$

Here *S* is a positive semidefinite matrix of coefficients. *λ* is the smoothing parameter which can be given by *β*. We then obtain the evaluated *β* vector, $\hat{\beta }$. The probability of metabolites being differential and compensated can be evaluated as

(8) $$\begin{array}{c} \hat{Y}=X\hat{\beta }. \end{array}$$

Finally, the combined global nonequivalence and bias (CGNB) score vector of a metabolite, *C*, can be calculated as

(9) $$\begin{array}{c} C=1-\hat{Y}. \end{array}$$

Thus, a high CGNB value represents a metabolite that is difficult to be identified in the profile and also not easily compensated for, which indicates that the metabolite should be paid more attention.

#### 2.2.4. Evaluating the Significance of Pathways

We used the Wallenius approximation [15] to evaluate the significance of pathways. This method is an extended version of the hypergeometric test, which involves weighted parameters. In MPINet, we assumed that the probability of metabolites being identified in the profile and being compensated for within a pathway differed from that of metabolites within other pathways. For each pathway, a weight can be calculated based on the CGNB scores of the metabolites within it. For pathways containing metabolites that are difficult to compensate for under dysregulated conditions and are difficult to identify in the profile, MPINet will enhance their competitiveness through the pathway-weight value. For a given pathway, the following values are required for this step: (i) the number of interesting metabolites (*n*); (ii) the number of background metabolites (*N*); (iii) the number of background metabolites annotated to this pathway (*m*_1_); (iv) the number of interesting metabolites annotated to this pathway (*g*); (v) the weight of this pathway (*w*_1_). First, the relative weight of the pathway is calculated as follows:

(10) $$\begin{array}{c} W=\frac{\left(1/\left|P\right|\right)\sum_{j\in P}\left(1-C_{j}\right)}{\left(1/K\right)\sum_{k=1}^{K}\left(\left(1/\left|P_{k}\right|\right)\sum_{r\in P_{k}}\left(1-C_{r}\right)\right)}, \end{array}$$

where *P* is the metabolite set of the pathway, |*P*| is the size of *P*, *j* is the metabolite in *P*, *C*_*j*_ is the CGNB score of the metabolite *j*, *K* is the number of pathways selected for analysis, *P*_*k*_ is the metabolite set in the *k*th pathway, |*P*_*k*_| is the size of *P*_*k*_, *r* is the metabolite in *P*_*k*_, and *C*_*r*_ is the CGNB score of the metabolite *r*. Finally, the significance *P*-value of this pathway can then be calculated as follows:

(11) P  value=1−∑x=0g−1((m1x)(m2n−x)) ×∫01(1−tw1/dx)x(1−tw2/dx)(n−x)dt,

where *d*_*x*_ = *w*_1_*(*m*_1_ − *x*) + *w*_2_*(*m*_2_ − (*n* − *x*)), *w*_1_ = *W* ^6^, *w*_2_ = 1, and *m*_2_ = *N* − *m*_1_.

## 3. Results

We firstly confirmed that the bias in metabolomics experimental technologies impacts on pathway identification. Then, we applied MPINet to four datasets, including a prostate cancer metastasis dataset, diabetes dataset 1, diabetes dataset 2, and a drug-sensitivity dataset. In the analysis of the prostate cancer metastasis dataset, we aimed to compare the effectiveness of MPINet and other currently popular methods, including the hypergeometric test (ORA), MSEA (SEA), and MetPA. We then applied MPINet to a different biological phenotype (diabetes) to identify novel diabetes-related pathways. Diabetes dataset 2 was used to demonstrate the reproducibility of MPINet. Finally, we applied MPINet to a drug-sensitivity dataset to test its validity with a completely different biological problem.

### 3.1. Bias in Metabolomics Experimental Technologies Impacts on Pathway Identification

These identified metabolites which were provided by the metabolomic technology are small in amount and not random selected. These identified metabolites in the profiles are usually well-studied metabolites which tend to have many functional partners in the network. This will lead to the bias that pathways contain many well-studied metabolites are likely to be inappropriately identified.

**Figure 2:**
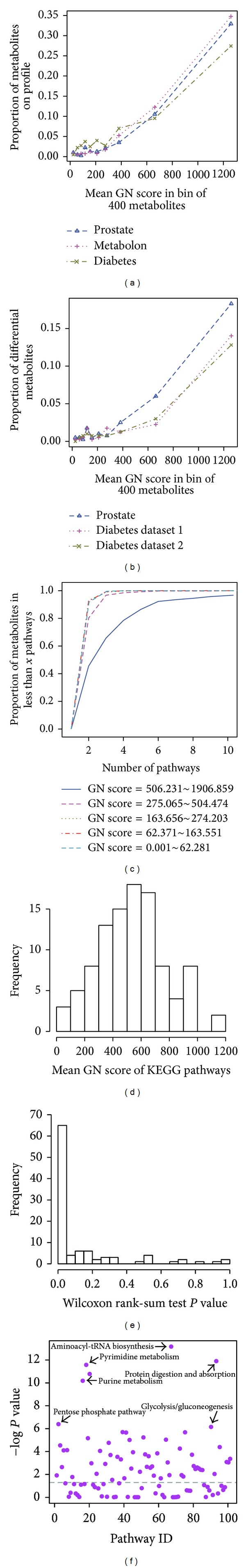
Validation of bias in metabolite pathway identification based on 101 pathways with more than five metabolites each. (a) The proportion of metabolites in the profile plotted against the mean GN score in a bin of 400 metabolites in the global human metabolite network across the three profiles. (b) The proportion of differential metabolites plotted across the three disease datasets. (c) Cumulative distribution of number of pathways associated with metabolites at a given GN score level. (d) Frequency of mean GN scores of metabolites in pathways. (e) *P* values for two-sided Wilcoxon's rank-sum test comparing the GN score of metabolites in the given pathway with that of the overall metabolites. (f) Scatter plot of pathway *P* value distributions. *P* values were calculated by one-sided Wilcoxon's rank-sum test comparing GN scores of metabolites in a given pathway with overall metabolites.

In order to validate the bias in metabolite pathway identification, we performed the following analysis. First, we validated our assumption that metabolites with high GN scores were more likely to be identified in profiles and thus be considered as differential (i.e., the two main factors are not independent). We calculated the GN scores of metabolites identified in three different metabolomic profiles and showed that metabolites identified in metabolomic profiles tended to have high GN scores (Figure 2(a)). Differential metabolites also tended to have high GN scores (Figure 2(b)). We then calculated the GN scores of metabolites in the network and inspected the number of pathways in which they participated. As expected, metabolites with high GN scores tended to participate in multiple pathways, while metabolites with low GN scores tended to reside in few pathways (i.e., pathway-specific metabolites) (Figure 2(c)).

For the pathways, we calculated the GN scores of all the metabolites in KEGG pathways and found a wide variation in the distribution of metabolite GN scores (Figure 2(d)). We then used Wilcoxon's rank-sum test to determine if the GN scores of metabolites within each pathway differed significantly from random. More than half of all pathways had significantly differential GN-score metabolites (*P* < 0.05, Figure 2(e)). We similarly analyzed all 3189 human pathways from ConsensusPathDB [21] and found consistent results (Figure S1). We also found that pathways including metabolites with high GN scores were usually fundamental pathways (Figure 2(f)). Overall, almost half of the analyzed pathways tended to contain metabolites with significantly high or low GN scores compared with random; however, the metabolites identified in the profiles and differential metabolites tended to have high GN scores (Figures 2(a) and 2(b)), suggesting that pathways containing many high-GN-score metabolites (i.e., usually fundamental pathways) were preferentially identified, while pathways containing many low-GN-score metabolites tended to be ignored by most currently used methods. A method that takes account of this bias is thus required.

### 3.2. Network-Based Metabolite Pathway Identification (MPINet) Can Effectively Identify Pathways Implicated in Prostate Cancer Metastasis

We determined the effectiveness of MPINet for identifying pathways associated with prostate cancer metastasis and compared the results with those of three other popular methods, including ORA (hypergeometric test), MSEA, and MetPA.

#### 3.2.1. Comparison of MPINet with ORA

**Figure 3:**
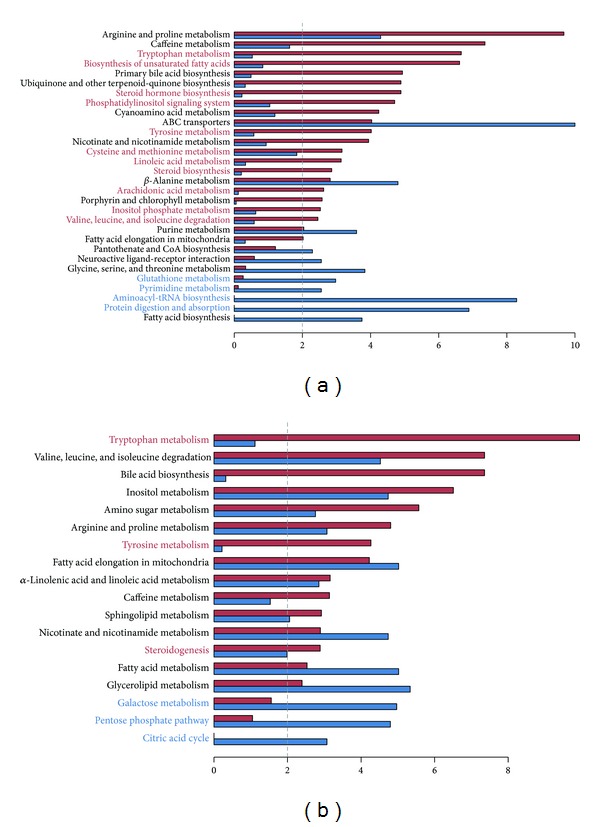
Comparisons between MPINet and other methods. *Y*-axis represents pathways, and *x*-axis is the −log10 transformation of FDR-values. Red bars represent pathway results identified by MPINet and blue bars represent the results of ORA (MSEA). Pathway names marked in red were uniquely identified by MPINet. Pathway names marked by blue were uniquely identified by ORA (MSEA). (a) MPINet versus ORA. (b) MPINet versus MSEA.

To demonstrate the advance of MPINet, we compared pathways identified by MPINet to those by ORA using a prostate cancer dataset. MPINet identified twenty-two significant pathways, associated with prostate cancer metastasis, with a strict false discovery rate (FDR) cut-off level (FDR < 0.01). Among these pathways, up to 14 were well documented to be implicated in cancer (detailed information is provided in supplementary Table S3). After applying the ORA method to the same prostate cancer dataset, we identified 12 significant pathways under the same strict cut-off level (FDR < 0.01). Compared with ORA, MPINet identified 18 unique pathways, 11 of which were reported to associate with metastatic cancer or cancer (pathway names marked red in Figure 3(a); Table S3). These results indicate the superior ability of MPINet to identify cancer-related pathways.

**Figure 4:**
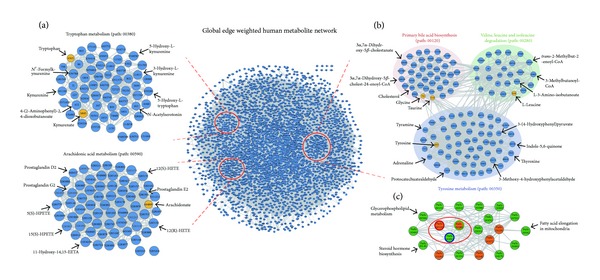
Global-weighted human metabolite network for several pathways identified by MPINet. The yellow node represents differential metabolites. Node size is proportional to the CGNB score of metabolites in (a) and (b). (a) Two metastatic prostate cancer-related pathways: “tryptophan metabolism” and “arachidonic acid metabolism.” (b) Three type 2 diabetes-related pathways: “primary bile acid biosynthesis,” “valine, leucine, and isoleucine degradation,” and “tyrosine metabolism.” (c) A global view of the interaction between the 21 type 2 diabetes-associated pathways. The edges between two pathways are displayed when the average GCS value between metabolite sets in the two pathways is greater than the median GCS value. Edge-line width is proportional to the average GCS value. Orange nodes represent pathways known to be related to type 2 diabetes. The red circle in the network corresponds to the three pathways in (b).

The most significant additional pathway identified by MPINet was the “tryptophan metabolism” pathway. MPINet yielded a FDR of 2.18*E* − 07. However, this pathway was not significant even at the 10% level in the ORA method (FDR = 0.29). The degradation of tryptophan mediated by indoleamine 2,3-dioxygenase can influence the tumoral immune response [26]. Recently, Opitz et al.'s study also found that kynurenine, which is derived from tryptophan through tryptophan-2,3-dioxygenase, can suppress antitumor immune responses and promote tumor-cell survival and motility [27]. Furthermore, it has been reviewed that the subregion of tryptophan metabolism pathway which starts from tryptophan was reported to be implicated with the cell proliferation of prostate cancer [28]. An inspection of this pathway showed that there were only three differential metabolites annotated in this pathway and two of which, tryptophan and kynurenate, were presented in the network (Figure 4(a) path: 00380). Kynurenate is a catabolite generated by kynurenine which was reported to promote the survival and motility process of tumor [27]. Recent studies found that the subregion of this pathway that converted tryptophan to kynurenate which includes tryptophan, N′-formylkynurenine, kynurenine, 4-(2-aminophenyl)-2,4-dioxobutanoate, and kynurenate is closely related with the tumor progression such as survival and motility (Figure S2) [27]. Interestingly, most of metabolites in this pathway had high CGNB scores, suggesting that they were pathway-specific and thus not easily detected in the metabolomic profile. These results suggest that the nondifferential metabolites in the subpath region from tryptophan to kynurenate, including N′-formylkynurenine, kynurenine, and 4-(2-aminophenyl)-2,4-dioxobutanoate (Figure 4(a) path: 00380), may also be associated with cancer. MPINet increased the competitiveness of the “tryptophan metabolism” pathway by integrative analysis of the metabolite network structure and metabolomic profile. However, ORA ignored this pathway because a few metabolites were annotated to this pathway. ORA analysis also ignored the nonequivalence of metabolites and the bias inherent in metabolite pathway analysis.

MPINet identified the “arachidonic acid metabolism” pathway with FDR = 0.0023. Most metabolites in this pathway had high CGNB scores (Figure 4(a): path: 00590), but surprisingly, only one interesting metabolite is annotated in this pathway. ORA analysis thus disregarded this pathway with a high FDR value 0.76. However, the arachidonic acid metabolism pathway has been reported to highly associate with the progression of prostate tumor [29]. Previous studies have shown that arachidonic acid can mediate the progression of prostate cancer metastasis to bone and affect the metabolism of cancer in bone stromal cells [30]. In addition, a wide range of studies have shown that arachidonic acid can affect the progression of malignant prostate cancer through metabolites produced in the COX and LOX processes [31–33]. These metabolites further influence many cancer invasion-related activities, including proliferation, apoptosis, and angiogenesis [31–33]. For example, Yang et al. [33] demonstrated that the LOX metabolite 12-HETE was important for the progression of prostate carcinoma.

#### 3.2.2. Comparison of MPINet with Other Methods

We also compared MPINet with MSEA and MetPA. The MSEA method identified 13 significant pathways in the prostate cancer dataset, under the strict cut-off value for significance (FDR < 0.01). Because MSEA uses the SMPDB pathways in the MSEA library as pathway databases, rather than KEGG, we also applied MPINet to the same pathway databases to ensure a fair comparison. MPINet identified 15 significant pathways (FDR < 0.01) when using SMPDB pathway databases. Most of the pathways (10 pathways) identified by MSEA were also included in the pathway list for MPINet (Figure 3(b)), and three pathways (marked red in Figure 3(b)) including “tryptophan metabolism,” “tyrosine metabolism,” and “steroidogenesis” pathway have been reported to be highly associated with cancer and were uniquely identified by MPINet. The tryptophan metabolism has been well documented in the literatures to be implicated with the progression of prostate cancer such as promoting the survival and motility of tumor cell and suppressing the antitumor immune response [27]. MSEA uses the whole list of metabolites in a profile and metabolite concentration changes. However, this quantitative information is unavailable for most metabolites. The “tryptophan metabolism” pathway, as discussed above that highly associates with prostate cancer, was also uniquely identified by MPINet in this comparison (Figure 3(b)). Inspection of this pathway shows that only two differential metabolites were identified in the profile, while no quantitative information was available for the other metabolites in the pathway (Figure 4(a): path 00380). In addition, MSEA treats each pathway as a simple metabolite set and ignores functional interactions among metabolites within the biological network. Two further pathways, “tyrosine metabolism” and “steroidogenesis,” are also reported to be associated with cancer [34, 35]. For example, the norepinephrine is closely related with tumor and the norepinephrine metabolism which has been reported to highly associate with the initiation and progression of tumor is a subprocess of the tyrosine metabolism pathway [34, 36, 37]. However, three pathways identified by MSEA, “galactose metabolism,” “pentose phosphate pathway,” and “citric acid cycle” (marked blue in Figure 3(b)), are more fundamental pathways.

We applied MetPA to the prostate cancer dataset. For fair comparison, 72 pathways in our KEGG pathway data, which were also in the 80 human pathway library of MetPA, were selected for comparison. The results of MetPA are given as impact scores, and we therefore compared pathway rank list from MPINet and MetPA. MPINet detected 15 pathways at a significance level of FDR < 0.01, based on this pathway dataset, up to nine of which are known to be related to cancer (Table 1). Arginine and proline metabolism is the most significant pathway identified by MPINet. Several studies have shown that arginine and proline metabolism regulated the immune responses and tumor growth and metastasis [38, 39]. We then examined their ranks in the MetPA pathway list. Among these nine pathways, the MPINet ranks of seven surpassed those in MetPA (marked by stars in Table 1). Six of these seven pathways were ranked >15 in MetPA. For example, the top ranked of the six pathways was the “tryptophan metabolism” pathway which is highly associated with the tumor progression, with a MetPA impact score of only 0.14 (rank 19). The impact score in MetPA is calculated as the normalized sum of importance measures of differential metabolites and also depends on the number of differential metabolites. However, as shown in Figure 4(a), most metabolites in this pathway were not identified in the profile. Furthermore, most of the metabolites in this pathway are pathway-specific (Figure 4(a)), suggesting that they are less likely to be compensated for by factors outside this pathway. MPINet paid more attention to this pathway by considering the global nonequivalence of metabolites and the bias associated with the experimental technology. MetPA, however, disregarded this pathway because of local importance measures and ignoring the bias.

### 3.3. Identifying Pathways Related to Type 2 Diabetes

#### 3.3.1. MPINet's Potential to Identify Novel Type 2 Diabetes-Related Pathways

We applied MPINet to a different phenotype and demonstrated its power to identify novel pathways potentially related to type 2 diabetes. We initially analyzed type 2 diabetes dataset 1. MPINet identified 21 pathways with FDR < 0.01 (Table S4) and up to six of which belonged to lipid metabolism, which has been reported to play an important role in the pathogenesis of type 2 diabetes, mainly through its influence on insulin resistance [40, 41]. Furthermore, lipid management represents a useful strategy for reducing vascular risk in patients with diabetes mellitus [42]. Among these six identified lipid-metabolism pathways, “primary bile acid biosynthesis,” “biosynthesis of unsaturated fatty acids,” and “synthesis and degradation of ketone bodies” have previously been reported to be associated with type 2 diabetes [43–46]. The most significant additional pathway related to type 2 diabetes was the “primary bile acid biosynthesis” pathway, with an FDR value of 1.45*E* − 05 (rank 3), compared with an FDR value of 0.216 (rank 34) yielded by ORA, and an impact value of only 0.07 (ranked 23) reported by MetPA. Most metabolites in this pathway tended to be pathway-specific and thus difficult to be identified (Figure 4(b)). Bile acid sequestration maintains lipid concentrations by mediating bile acid metabolism, and bile acid has the ability to influence systemic glucose metabolism [43]. Furthermore, Kobayashi et al. [44] also found that bile acids impacted on glucose metabolism and proposed the bile acid metabolism pathway as a novel potential therapeutic target pathway in type 2 diabetes. Therefore, bile acid sequestrants such as cholestyramine have been developed for the treatment of type 2 diabetes [47]. Biosynthesis of unsaturated fatty acids pathway is also highly associated with type 2 diabetes. The animal model experimental study of Krishna Mohan and Das suggested that polyunsaturated fatty acids have the ability of suppressing the occurrence of diabetes mellitus induced by chemical [48]. Moreover, the similar conclusion was review in that elevating the consumption of n-3 long chain polyunsaturated fatty acids may prevent type 2 diabetes and increasing evidences support this conclusion [45]. Three other possible novel type 2 diabetes-related lipid-metabolism pathways were identified by MPINet (FDR < 0.01), including “steroid hormone biosynthesis,” “fatty acid elongation in mitochondria,” and “glycerophospholipid metabolism.”

MPINet also identified some additional pathways, including “valine, leucine, and isoleucine degradation,” “valine, leucine, and isoleucine biosynthesis,” and “histidine metabolism,” which are related to type 2 diabetes [49–51]. The most significant of these was the “valine, leucine, and isoleucine degradation” pathway, which was identified by MPINet with an FDR value of 3.33*E* − 05 (rank 4), compared with an FDR value of 0.042 (rank 14) in the ORA method, and an impact score of only 0.09 in MetPA (rank 19). Inspection of this pathway showed that it included many metabolites with high CGNB scores (Figure 4(b)). Leucine-mediated mTORC1 signaling activation and mTORC1 together with its downstream target are important regulators of insulin resistance, which is a key feature of type 2 diabetes [50, 51]. The “histidine metabolism” pathway may be implicated with type 2 diabetes because histidine has the ability to suppress the production of hepatic glucose, and Kimura et al. suggest that histidine or the suppression of hepatic glucose production mediated by histidine is a potential therapeutic target of type 2 diabetes [49].

Finally, we investigated the crosstalk between the 21 pathways identified by MPINet with a significance level of FDR < 0.01. Interestingly, the additional “tyrosine metabolism” (path: 00350) pathway was closely connected with “primary bile acid biosynthesis” (path: 00120) and “valine, leucine, and isoleucine degradation” (path: 00280) in the network (Figure 4(b)). These results indicate that this pathway may also be associated with type 2 diabetes. Furthermore, several pathways not associated with type 2 diabetes in the literature were found to interact closely with well-reported type 2 diabetes-related pathways (Figure 4(c)).

#### 3.3.2. Reproducibility of MPINet

The type 2 diabetes dataset 2 was used to evaluate the reproducibility of MPINet. We selected 66 differential metabolites from the data of Suhre et al. (see Section 2). Only 30.7% of metabolites in diabetes dataset 1 overlapped with those in diabetes dataset 2. However, up to 20 (76.9%) pathways identified in diabetes dataset 2 overlapped with diabetes dataset 1 (FDR < 0.05). The overlapped pathways were highly significant *P* = 2.36*e* − 11, hypergeometric test. Some critical pathways such as “valine, leucine, and isoleucine degradation,” “valine, leucine, and isoleucine biosynthesis,” “primary bile acid biosynthesis,” and “histidine metabolism,” which are implicated in the progression of type 2 diabetes, were also identified in diabetes dataset 2. These results suggest that MPINet performs reliably in metabolomics pathway identification.

### 3.4. Identifying Drug-Sensitivity-Associated Pathways

**Figure 5:**
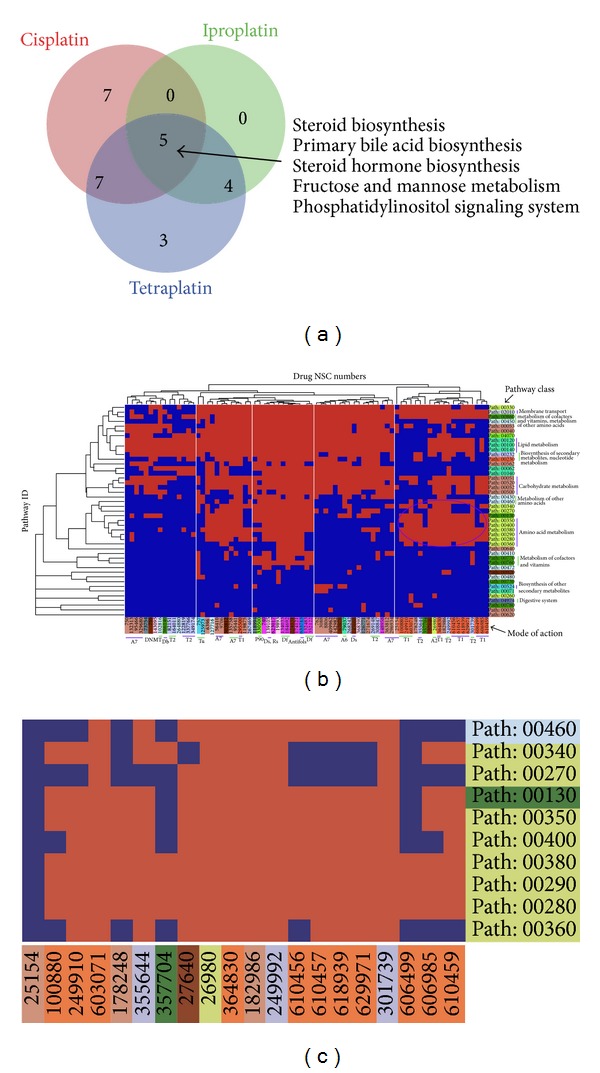
Identification of drug-sensitivity-related pathways. (a) Venn plot of pathways related to platinum-based-drug sensitivity. (b) Hierarchical clustering of drugs and sensitivity-related pathways. The corresponding cell was colored orange if the pathway was significantly associated with drug sensitivity (FDR < 0.01). (c) Zoom-in plot of the circle region in (b).

We investigated the effectiveness of MPINet for identifying drug-sensitivity-related pathways. Platinum-based drugs are widely used to treat many types of tumors, such as colon cancer, lung cancer, breast cancer, and ovarian cancer. However, resistance to platinum-based drugs is a major bottleneck in cancer therapy. The identification of consistent pathways relevant to platinum-based drug sensitivity is therefore of considerable importance. We analyzed the platinum-based drugs tetraplatin, iproplatin, and cisplatin and identified 55, 17, and 44 significant metabolites (FDR < 60%) associated with these three drugs, respectively. Through inputting these metabolites, MPINet identified 19, 9, and 19 significant pathways as drug-sensitivity-related pathways, respectively (FDR < 0.01). As we expected, most of the pathways identified by MPINet were shared by at least two of the three drugs (Figure 5(a)). These results indicate that MPINet was able to identify highly consistent pathways associated with the sensitivities of these three platinum-based drugs. MPINet identified five sensitivity-related pathways shared by all three drugs, including the three lipid metabolism pathways, “steroid biosynthesis,” “primary bile acid biosynthesis,” and “steroid hormone biosynthesis.” Some previous studies have shown that lipid metabolism may influence the effects of platinum-based drugs [52], and Hendrich and Michalak [53] suggested that membrane-lipid composition might be associated with multidrug resistance. These results demonstrate the ability of MPINet to identify effectively pathways associated with platinum-based drug sensitivity. Furthermore, we expansively applied MPINet to all 121 drugs (see Section 2) and identified the sensitivity pathways for each drug. From a global point of view, drugs assigned to the same mode of action tended to be associated with sensitivity pathways from the same class (Figure 5(b)). For example, amino acid metabolic pathways tended to be related to topoisomerase I inhibitor sensitivity (Figure 5(c)). Overall, these results indicate that MPINet provides a robust and effective method for identifying drug-sensitivity pathways.

## 4. Conclusion and Discussion

The major innovations of MPINet include the simultaneous evaluation of the global nonequivalence of metabolites based on the global network structure and consideration of the bias associated with metabolomic experimental technology. From a biological perspective, dysfunctions of metabolites with strong functional-interaction partners are more likely to be compensated for than those with weak functional-interaction partners. Taking account of these functional interactions (network structure) to evaluate the nonequivalence of metabolites will help to improve the power of pathway identification. From the experimental technology perspective, the low coverage of metabolomics [9] and metabolite identification bias can have a great impact on subsequent pathway analysis (Figure 2). Based on these considerations, we developed a novel metabolite pathway identification method that takes account of the above aspects by allowing the integrated analysis of functional interactions between metabolites in the global biological network and the character of the metabolomic profile. We demonstrated the effectiveness and reproducibility of MPINet and its wide applicability across different application fields by analyzing several real datasets.

Metabolites involved in fundamental pathways have often been well studied and tend to be easily detected in the profile (Figure 2). These fundamental pathways may thus be preferentially identified if this bias is not adjusted for. In contrast, pathways that contain many pathway-specific metabolites (i.e., metabolites with low GN score) (specific pathways) will be more easily ignored. However, specific pathways have often been reported to be associated with diseases. If the metabolites in a pathway tend to be pathway-specific, dysregulation signals will be amplified and the entire pathway may even be shut down. An example of this is the arachidonic acid metabolism pathway, which includes many metabolites with high CGNB scores (Figure 4(a)). The metabolites are influenced by dysfunctional arachidonic acid and may not be easily compensated for by functional-interaction partners. MPINet pays more attention to pathway-specific metabolites by considering the nonequivalence of metabolites from a global perspective; MPINet increases the competitiveness of pathways containing many metabolites with high CGNB scores and decreases the competitiveness of pathways that contain many well-studied metabolites.

Compared with other current methods such as ORA, MSEA, and MetPA, MPINet can integrate not only metabolites from metabolomics experiments, but also the global metabolite network structure to enhance the ability of pathway identification. One of the limitations of current metabolomics technology is that many metabolites cannot be identified. MPINet utilizes the global metabolite functional network to compensate for the lack of information. Thus, in the case of pathways with weak signals from the annotation number point of view, MPINet can also identify these by considering the additional global network structure. MSEA can also detect some weak pathways [54, 55] by considering the additional nondifferential metabolites in the metabolomic profile; however, compared with MSEA, the additional information in MPINet is based on global functional level rather than on the concentration of a single metabolite. Moreover, MPINet only requires simple input consisting of a list of interesting metabolites. Taken together, MPINet has the potential to complement ORA, MSEA, and MetPA and may also be a useful tool in subsequent studies, such as studies of disease classification based on biologically meaningful pathways [56].

## Supplementary Material

Figure S1: Validation of bias via analyzing the 3189 human pathways in the ConsensusPathDB database.
Figure S2: Tryptophan metabolism pathway identified by MPINet, in which the differential metabolites of prostate cancer metastasis were annotated.
Supplementary text: Detailed description of calculating the global connection strength (GCS).
Table S1: The type 2 diabetes associated metabolites from text mining and HMDB database.
Table S2: The detailed information of human metabolite background from five databases.
Table S3: The statistically significant pathways identified by MPINet method for differential metabolites from metastatic prostate cancer dataset (FDR<0.01).
Table S4: The twenty-one pathways identified by MPINet method for interesting metabolites from the type 2 diabetes dataset 1 (FDR<0.01).

## Figures and Tables

**Table 1:** Top-ranked 15 pathways in MPINet and their ranks in MetPA.

Pathway name	FDR-N	R-P	I-P
**Arginine and proline metabolism∗**	**8.15*E* − 09**	**6**	**0.21**
Caffeine metabolism	1.16*E* − 07	12	0.17
**Tryptophan metabolism∗**	**5.75*E* − 07**	**17**	**0.14**
Primary bile acid biosynthesis	2.54*E* − 05	17	0.14
Ubiquinone and other terpenoid-quinone biosynthesis	2.65*E* − 05	33	0.05
**Steroid hormone biosynthesis∗**	**2.65*E* − 05**	**38**	**0.03**
Cyanoamino acid metabolism	0.00019	12	0.17
**Tyrosine metabolism∗**	**0.00034**	**34**	**0.04**
Nicotinate and nicotinamide metabolism	0.0004	26	0.07
**Linoleic acid metabolism**	**0.0012**	**1**	**0.62**
**Cysteine and methionine metabolism**	**0.0039**	**9**	**0.19**
**Arachidonic acid metabolism∗**	**0.0039**	**—**	**—**
Porphyrin and chlorophyll metabolism	0.0043	34	0.04
**Inositol phosphate metabolism∗**	**0.007**	**17**	**0.14**
**Valine, leucine, and isoleucine degradation∗**	**0.0081**	**26**	**0.07**

FDR-N: FDR values of pathways in MPINet; R-P and I-P: ranks and impact scores for MetPA. Bold pathways have been well reported to be related with cancer. Ranks of pathways marked by asterisk in MPINet surpass that in MetPA.
