# Supplementary material for: MPINet: Metabolite Pathway Identification via Coupling of Global Metabolite Network Structure and Metabolomic Profile
Source: Biomed Res Int. 2014 Jun 25;2014:325697. doi: 10.1155/2014/325697 (PMC4095715; doi:10.1155/2014/325697)
Supplement: Supplementary file 1 — Figure S1: Validation of bias via analyzing the 3189 human pathways in the ConsensusPathDB database. Figure S2: Tryptophan metabolism pathway identified by MPINet, in which the differential metabolites of prostate cancer metastasis were annotated. Supplementary text: Detailed description of calculating the global connection strength (GCS). Table S1: The type 2 diabetes associated metabolites from text mining and HMDB database. Table S2: The detailed information of human metabolite background from five databases. Table S3: The statistically significant pathways identified by MPINet method for differential metabolites from metastatic prostate cancer dataset (FDR<0.01). Table S4: The twenty-one pathways identified by MPINet method for interesting metabolites from the type 2 diabetes dataset 1 (FDR<0.01). [file 325697.f1.doc]

**Supplementary Data**

**Supplementary Figures**


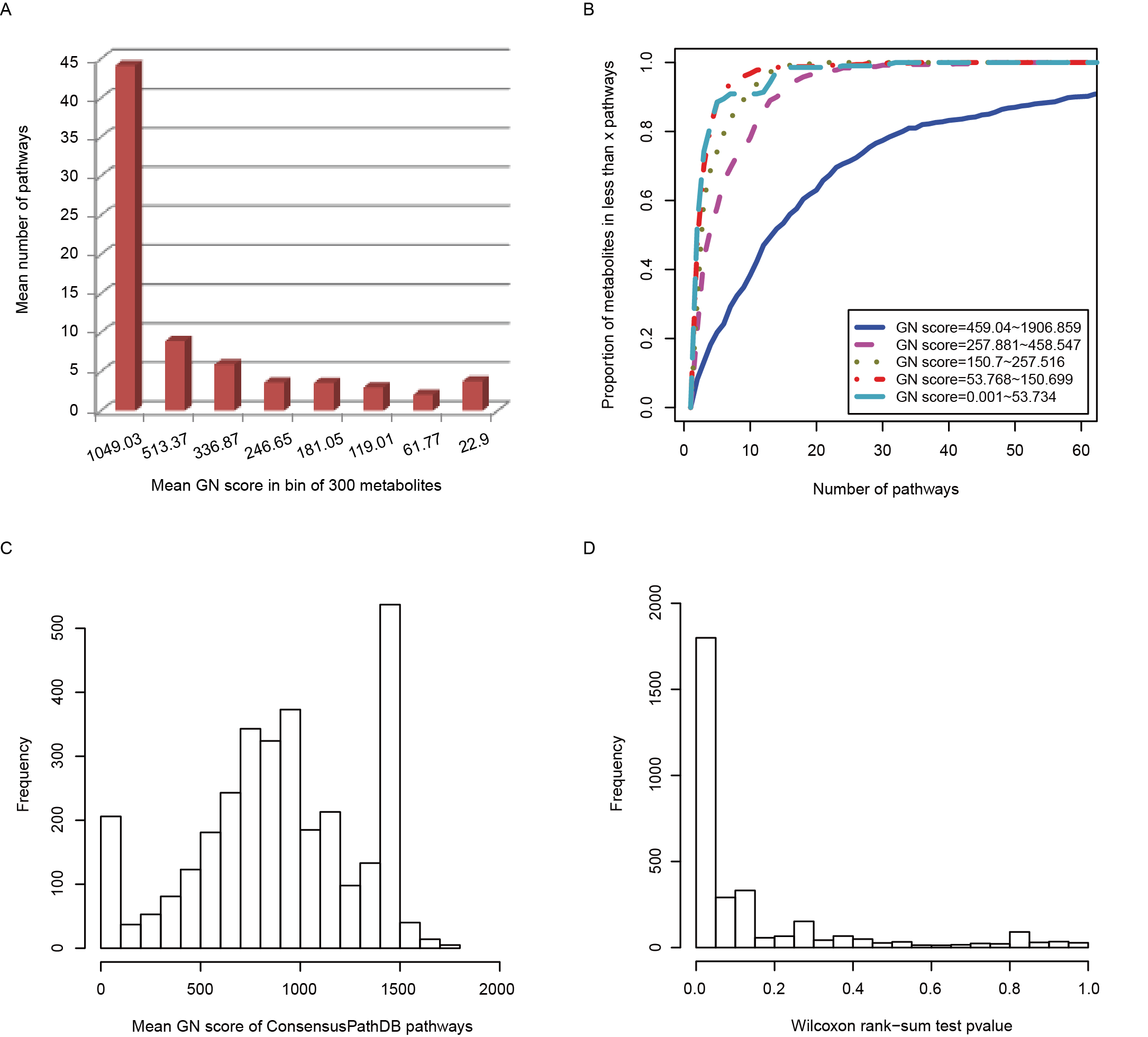
**Figure S1.** Validation of bias via analyzing the 3189 human pathways in the ConsensusPathDB database. (A) The mean number of pathways associated with metabolites at different mean GN score levels in 300 bin size. X-axis is the mean GN score of metabolites in a bin of 300 metabolites, the y -axis represents the average number of pathways which are participated by metabolites in the corresponding bin. (B) Cumulative distribution of number of pathways associated with metabolites at a given GN score level. (C) The frequency of mean GN score of metabolites in ConsensusPathDB pathways. (D) P-values for the two-sided wilcoxon rank-sum test comparing the GN score of metabolites in the given pathway with the overall metabolites in these 3189 pathways.

**
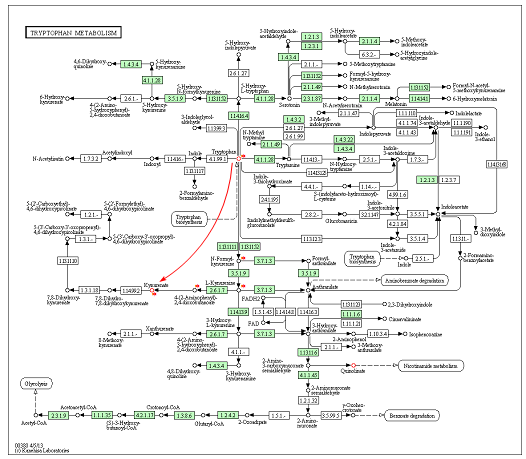
**

**Figure S2.** Tryptophan metabolism pathway identified by MPINet, in which the differential metabolites of prostate cancer metastasis were annotated. Nodes marked with asterisk belong to the sub region that converted tryptophan to kynurenate which includes tryptophan, N’-formylkynurenine, kynurenine, 4-(2-aminophenyl)-2,4-dioxobutanoate and kynurenate. Differential metabolites were marked with red node borders.

**Supplementary text**

**Calculating the global connection strength**

We calculated the global connection strength (GCS) measure value between two nodes in the network according to the modified version of the SOC measure in the study of Campbell *et al* . For example, we calculated the global connection strength between node i and node j in the network, the steps are as follows:

1. The edge weight from the primary network were divided by 1000, and thus the weight were ranged from 0 to 1,
2. Then the edge weight obtained from (1) were subtracted by 1,
3. Find the shortest paths and the shortest path length of the two nodes which considered the processed weight obtained from (2), if i=j, we assigned 0 as their global connection strength,
4. The shortest path value were given to the nodes in the shortest path,
5. Delete the nodes on the shortest paths excluding node i and j , delete the edge between them if i and j are direct connected,
6. Repeat the step (3)-(5) if node i and j were connected, otherwise turn into next step,
7. the values that assigned to the above nodes were divided by 1 as its weight, for these nodes that did not assign a value we give 0 as its weight,
8. Finally, sum the weight of the nodes in network as the GCS value between node i and node j in the global edge weighted human metabolite network.

Higher GCS value indicates stronger functional interactions between the metabolite pair (i.e. the connected path between them tend to be more and shorter in the global metabolite network).

**Supplementary tables**

**Table S1:** the type 2 diabetes associated metabolites from text mining and their sources

| Metabolites | Sources (references) |
| --- | --- |
| isoleucine | Wang et al. |
| leucine | Wang et al. |
| valine | Wang et al. |
| tyrosine | Wang et al. |
| phenylalanine | Wang et al. |
| Ornithine | Wang et al. |
| Tryptophan | Wang et al. |
| Proline | Wang et al. |
| Histidine | Wang et al. |
| Cotinine | Wang et al. |
| 5'-Adenosylhomocysteine | Wang et al. |
| Alanine | Wang et al. |
| lactate | Zeng et al. |
| a-hydroxyisobutyric acid | Zeng et al. |
| phosphate | Zeng et al. |
| 1-monopalmitin | Zeng et al. |
| 1-monostearin | Zeng et al. |
| 2-ketoisocaproic acid | Zeng et al. |
| Alanine | Zeng et al. |
| b-hydroxybutyric acid | Zeng et al. |
| Leucine | Zeng et al. |
| Isoleucine | Zeng et al. |
| Serine | Zeng et al. |
| Pyroglutamic acid | Zeng et al. |
| Palmitic acid | Zeng et al. |
| Oleic acid | Zeng et al. |
| Stearic acid | Zeng et al. |
| Arachidonic acid | Zeng et al. |
| palmitic acid | Yi et al. |
| stearic acid | Yi et al. |
| oleic acid | Yi et al. |
| glycine | Wang-Sattler et al. |
| lysophosphatidylcholine (LPC) | Wang-Sattler et al. |
| acetylcarnitine | Wang-Sattler et al. |
| triglycerides | Rhee et al. |
| HDL cholesterol | Rhee et al. |
| hexose | Floegel et al. |
| phenylalanine | Floegel et al. |
| diacyl-phosphatidylcholines | Floegel et al. |
| glycine | Floegel et al. |
| sphingomyelin | Floegel et al. |
| acyl-alkyl-phosphatidylcholines | Floegel et al. |
| lysophosphatidylcholine | Floegel et al. |
| Glycerophosphate | Daimon et al. |
| Octanoate | Daimon et al. |
| Glycerophosphorylcholine | Daimon et al. |
| Threonine | Daimon et al. |
| Arginine | Daimon et al. |
| phenylalanine | Daimon et al. |
| Methionine Sulfoxide | Daimon et al. |
| Hexanoate | Daimon et al. |
| tyrosine | Daimon et al. |
| Heptanoate | Daimon et al. |
| Serine | Daimon et al. |
| Histidine | Daimon et al. |
| 2-Aminobutanoate | Daimon et al. |
| Acetohydroxamate | Daimon et al. |
| lactate | Daimon et al. |
| leucine | Daimon et al. |
| Choline | Daimon et al. |
| Proline | Daimon et al. |
| Asparagine | Daimon et al. |
| Lysine | Daimon et al. |
| Alanine | Daimon et al. |
| Hypoxanthine | Daimon et al. |
| Taurine | Daimon et al. |
| Ornithine | Daimon et al. |
| (S)-3-Hydroxyisobutyric acid | HMDB |
| Acetoacetic acid | HMDB |
| Acetone | HMDB |
| 1-Butanol | HMDB |
| 3-Hydroxybutyric acid | HMDB |
| Dimethylamine | HMDB |
| Glycerol | HMDB |
| Pyruvaldehyde | HMDB |
| Scyllitol | HMDB |
| S-Adenosylmethionine | HMDB |
| Uric acid | HMDB |
| Estriol | HMDB |
| D-Glucose | HMDB |
| Dodecanedioic acid | HMDB |
| Fructosamine | HMDB |
| Chromium | HMDB |
| Hyaluronan | HMDB |
| 4-Heptanone | HMDB |
| D-Lactic acid | HMDB |
| 1,5-Anhydrosorbitol | HMDB |
| 3-Methylhistidine | HMDB |
| 8-Hydroxyguanine | HMDB |
| 1-Methylhistidine | HMDB |
| (R)-3-Hydroxybutyric acid | HMDB |
| D-Fructose | HMDB |
| L-Carnitine | HMDB |

**Table S2. The human metabolite background from five databases.**

| HMDB and KEGG | Reactome | SMPDB | MSEA | Total (unique) |
| --- | --- | --- | --- | --- |
| 4703 | 809 | 761 | 1361 | 4994 |

**Table S3.** The statistically significant pathways identified by MPINet method for differential metabolites from metastatic prostate cancer data set (FDR<0.01).

| pathwayId | pathwayName | pvalue | fdr | weight | Possible relation to the cancer | Reference |
| --- | --- | --- | --- | --- | --- | --- |
| path:00330 | Arginine and proline metabolism | 3.53E-12 | 2.12E-10 | 0.22 | regulation of immune responses and tumor growth and metastasis |  |
| path:00232 | Caffeine metabolism | 1.46E-09 | 4.37E-08 | 0.0055 | ----- | ----- |
| path:00380 | Tryptophan metabolism | 1.09E-08 | 2.18E-07 | 0.0027 | mediate proliferation and tumoral immune resistance mechanism |  |
| path:01040 | Biosynthesis of unsaturated fatty acids | 1.62E-08 | 2.43E-07 | 0.0051 | Influence prostate cancer metastasis and suppression of mTOR Signaling |  |
| path:00120 | Primary bile acid biosynthesis | 9.62E-07 | 1.15E-05 | 0.0015 | ----- | ----- |
| path:00130 | Ubiquinone and other terpenoid-quinone biosynthesis | 1.32E-06 | 1.29E-05 | 0.0011 | ----- | ----- |
| path:00140 | Steroid hormone biosynthesis | 1.50E-06 | 1.29E-05 | 0.00093 | Stimulate human prostate cancer progression and metastasis; |  |
| path:04070 | Phosphatidylinositol signaling system | 2.61E-06 | 1.96E-05 | 0.0085 | Regulation of cell survival, proliferation and growth and modulate the PI3K pathway |  |
| path:00460 | Cyanoamino acid metabolism | 8.61E-06 | 5.74E-05 | 0.065 | ----- | ----- |
| path:02010 | ABC transporters | 1.55E-05 | 9.27E-05 | 4.33 | Cell migration, invasion and metastasis |  |
| path:00350 | Tyrosine metabolism | 1.75E-05 | 9.55E-05 | 0.035 | Cancer therapy |  |
| path:00760 | Nicotinate and nicotinamide metabolism | 2.29E-05 | 0.00011 | 0.065 | ----- | ---- |
| path:00270 | Cysteine and methionine metabolism | 0.00014 | 0.00068 | 0.49 | Metabolites increase the ability to predict aggressive prostate cancer and infunce prostate cancer progression |  |
| path:00591 | Linoleic acid metabolism | 0.00017 | 0.00073 | 0.00036 | Stimulate growth of prostate cancer cell; promote proliferation and migration of PC-3 cells |  |
| path:00100 | Steroid biosynthesis | 0.00034 | 0.0013 | 0.00037 | High levels of cholesterol in PCa bone metastases; therapy of metastatic prostate cancer |  |
| path:00410 | beta-Alanine metabolism | 0.00040 | 0.0015 | 2.60 | ----- | ---- |
| path:00590 | Arachidonic acid metabolism | 0.00067 | 0.0023 | 0.00048 | altered immune response to cancer cells and modulation of inflammation, proliferation,  apoptosis, metastasis, and angiogenesis; |  |
| path:00860 | Porphyrin and chlorophyll metabolism | 0.00079 | 0.0026 | 0.00036 | ----- |  |
| path:00562 | Inositol phosphate metabolism | 0.00094 | 0.0029 | 0.080 | Related with cell survival, proliferation and growth in cancer and regulation of antitumor activity |  |
| path:00280 | Valine, leucine and isoleucine degradation | 0.0011 | 0.0035 | 0.076 | stimulate cell growth ,cell cycle progression by activation of mTOR signaling cascade and associated with apoptosis of tumour cells |  |
| path:00230 | Purine metabolism | 0.0031 | 0.0090 | 1.90 | treatment of cancer |  |
| path:00062 | Fatty acid elongation in mitochondria | 0.0034 | 0.0095 | 0.0069 | ----- | ---- |

**TableS4.** The twenty-one pathways identified by MPINet method for interesting metabolites from the type 2 diabetes data set 1 (FDR<0.01).

| pathwayId | pathwayName | pvalue | fdr |
| --- | --- | --- | --- |
| path:00460 | Cyanoamino acid metabolism | 6.14E-08 | 3.56E-06 |
| path:00860 | Porphyrin and chlorophyll metabolism | 2.88E-07 | 8.36E-06 |
| path:00120 | Primary bile acid biosynthesis | 7.50E-07 | 1.45E-05 |
| path:00280 | Valine, leucine and isoleucine degradation | 2.41E-06 | 3.33E-05 |
| path:01040 | Biosynthesis of unsaturated fatty acids | 2.87E-06 | 3.33E-05 |
| path:02010 | ABC transporters | 1.57E-05 | 0.000152 |
| path:00310 | Lysine degradation | 4.04E-05 | 0.000335 |
| path:00450 | Selenoamino acid metabolism | 0.00026 | 0.001774 |
| path:00340 | Histidine metabolism | 0.000275 | 0.001774 |
| path:00350 | Tyrosine metabolism | 0.000332 | 0.001926 |
| path:00290 | Valine, leucine and isoleucine biosynthesis | 0.000382 | 0.002016 |
| path:00072 | Synthesis and degradation of ketone bodies | 0.000506 | 0.002444 |
| path:04070 | Phosphatidylinositol signaling system | 0.000788 | 0.003508 |
| path:00130 | Ubiquinone and other terpenoid-quinone biosynthesis | 0.000847 | 0.003508 |
| path:00140 | Steroid hormone biosynthesis | 0.001098 | 0.004245 |
| path:00400 | Phenylalanine, tyrosine and tryptophan biosynthesis | 0.001419 | 0.005145 |
| path:00520 | Amino sugar and nucleotide sugar metabolism | 0.001762 | 0.006013 |
| path:00380 | Tryptophan metabolism | 0.002254 | 0.007262 |
| path:00062 | Fatty acid elongation in mitochondria | 0.002518 | 0.007375 |
| path:00330 | Arginine and proline metabolism | 0.002543 | 0.007375 |
| path:00564 | Glycerophospholipid metabolism | 0.00302 | 0.00834 |

**References**

1. Campbell, C., Thakar, J. and Albert, R. (2011) Network analysis reveals cross-links of the immune pathways activated by bacteria and allergen. *Phys Rev E Stat Nonlin Soft Matter Phys*, **84**, 031929.

2. Wang, T.J., Larson, M.G., Vasan, R.S., Cheng, S., Rhee, E.P., McCabe, E., Lewis, G.D., Fox, C.S., Jacques, P.F., Fernandez, C. *et al.* (2011) Metabolite profiles and the risk of developing diabetes. *Nat Med*, **17**, 448-453.

3. Zeng, M., Che, Z., Liang, Y., Wang, B., Chen, X., Li, H., Deng, J. and Zhou, Z. (2009) GC–MS Based Plasma Metabolic Profiling of Type 2 Diabetes Mellitus. *Chromatographia*, **69**, 941-948.

4. Yi, L.Z., He, J., Liang, Y.Z., Yuan, D.L. and Chau, F.T. (2006) Plasma fatty acid metabolic profiling and biomarkers of type 2 diabetes mellitus based on GC/MS and PLS-LDA. *FEBS Lett*, **580**, 6837-6845.

5. Wang-Sattler, R., Yu, Z., Herder, C., Messias, A.C., Floegel, A., He, Y., Heim, K., Campillos, M., Holzapfel, C., Thorand, B. *et al.* (2012) Novel biomarkers for pre-diabetes identified by metabolomics. *Mol Syst Biol*, **8**, 615.

6. Rhee, E.P., Cheng, S., Larson, M.G., Walford, G.A., Lewis, G.D., McCabe, E., Yang, E., Farrell, L., Fox, C.S., O’Donnell, C.J. *et al.* (2011) Lipid profiling identifies a triacylglycerol signature of insulin resistance and improves diabetes prediction in humans. *Journal of Clinical Investigation*, **121**, 1402-1411.

7. Floegel, A., Stefan, N., Yu, Z., Muhlenbruch, K., Drogan, D., Joost, H.G., Fritsche, A., Haring, H.U., Hrabe de Angelis, M., Peters, A. *et al.* (2013) Identification of serum metabolites associated with risk of type 2 diabetes using a targeted metabolomic approach. *Diabetes*, **62**, 639-648.

8. Daimon, M., Soga, T., Hozawa, A., Oizumi, T., Kaino, W., Takase, K., Karasawa, S., Jimbu, Y., Wada, K., Kameda, W. *et al.* (2012) Serum glycerophosphate levels are increased in Japanese men with type 2 diabetes. *Intern Med*, **51**, 545-551.

9. Wishart, D.S., Tzur, D., Knox, C., Eisner, R., Guo, A.C., Young, N., Cheng, D., Jewell, K., Arndt, D., Sawhney, S. *et al.* (2007) HMDB: the Human Metabolome Database. *Nucleic Acids Res*, **35**, D521-526.

10. Lind, D.S. (2004) Arginine and cancer. *J Nutr*, **134**, 2837S-2841S; discussion 2853S.

11. Bronte, V. and Zanovello, P. (2005) Regulation of immune responses by L-arginine metabolism. *Nat Rev Immunol*, **5**, 641-654.

12. Uyttenhove, C., Pilotte, L., Theate, I., Stroobant, V., Colau, D., Parmentier, N., Boon, T. and Van den Eynde, B.J. (2003) Evidence for a tumoral immune resistance mechanism based on tryptophan degradation by indoleamine 2,3-dioxygenase. *Nat Med*, **9**, 1269-1274.

13. Opitz, C.A., Litzenburger, U.M., Sahm, F., Ott, M., Tritschler, I., Trump, S., Schumacher, T., Jestaedt, L., Schrenk, D., Weller, M. *et al.* (2011) An endogenous tumour-promoting ligand of the human aryl hydrocarbon receptor. *Nature*, **478**, 197-203.

14. Siddiqui, E.J., Thompson, C.S., Mikhailidis, D.P. and Mumtaz, F.H. (2005) The role of serotonin in tumour growth (review). *Oncol Rep*, **14**, 1593-1597.

15. Brown, M.D., Hart, C., Gazi, E., Gardner, P., Lockyer, N. and Clarke, N. (2010) Influence of omega-6 PUFA arachidonic acid and bone marrow adipocytes on metastatic spread from prostate cancer. *Br J Cancer*, **102**, 403-413.

16. Shahidi, F. and Miraliakbari, H. (2004) Omega-3 (n-3) fatty acids in health and disease: Part 1--cardiovascular disease and cancer. *J Med Food*, **7**, 387-401.

17. Brown, M.D., Hart, C.A., Gazi, E., Bagley, S. and Clarke, N.W. (2006) Promotion of prostatic metastatic migration towards human bone marrow stoma by Omega 6 and its inhibition by Omega 3 PUFAs. *Br J Cancer*, **94**, 842-853.

18. Berquin, I.M., Edwards, I.J. and Chen, Y.Q. (2008) Multi-targeted therapy of cancer by omega-3 fatty acids. *Cancer Lett*, **269**, 363-377.

19. Friedrichs, W., Ruparel, S.B., Marciniak, R.A. and deGraffenried, L. (2011) Omega-3 fatty acid inhibition of prostate cancer progression to hormone independence is associated with suppression of mTOR signaling and androgen receptor expression. *Nutr Cancer*, **63**, 771-777.

20. Larsson, S.C., Kumlin, M., Ingelman-Sundberg, M. and Wolk, A. (2004) Dietary long-chain n-3 fatty acids for the prevention of cancer: a review of potential mechanisms. *Am J Clin Nutr*, **79**, 935-945.

21. Ricke, W.A., Ishii, K., Ricke, E.A., Simko, J., Wang, Y., Hayward, S.W. and Cunha, G.R. (2006) Steroid hormones stimulate human prostate cancer progression and metastasis. *Int J Cancer*, **118**, 2123-2131.

22. Wang, Y., Sudilovsky, D., Zhang, B., Haughney, P.C., Rosen, M.A., Wu, D.S., Cunha, T.J., Dahiya, R., Cunha, G.R. and Hayward, S.W. (2001) A human prostatic epithelial model of hormonal carcinogenesis. *Cancer Res*, **61**, 6064-6072.

23. Hayward, S.W., Wang, Y., Cao, M., Hom, Y.K., Zhang, B., Grossfeld, G.D., Sudilovsky, D. and Cunha, G.R. (2001) Malignant transformation in a nontumorigenic human prostatic epithelial cell line. *Cancer Res*, **61**, 8135-8142.

24. Ricke, E.A., Williams, K., Lee, Y.F., Couto, S., Wang, Y., Hayward, S.W., Cunha, G.R. and Ricke, W.A. (2012) Androgen hormone action in prostatic carcinogenesis: stromal androgen receptors mediate prostate cancer progression, malignant transformation and metastasis. *Carcinogenesis*, **33**, 1391-1398.

25. Yang, W. and Levine, A.C. (2011) Androgens and prostate cancer bone metastases: effects on both the seed and the soil. *Endocrinol Metab Clin North Am*, **40**, 643-653, x.

26. Bunney, T.D. and Katan, M. (2010) Phosphoinositide signalling in cancer: beyond PI3K and PTEN. *Nat Rev Cancer*, **10**, 342-352.

27. Fletcher, J.I., Haber, M., Henderson, M.J. and Norris, M.D. (2010) ABC transporters in cancer: more than just drug efflux pumps. *Nat Rev Cancer*, **10**, 147-156.

28. Eisenhofer, G., Kopin, I.J. and Goldstein, D.S. (2004) Catecholamine metabolism: a contemporary view with implications for physiology and medicine. *Pharmacol Rev*, **56**, 331-349.

29. Sreekumar, A., Poisson, L.M., Rajendiran, T.M., Khan, A.P., Cao, Q., Yu, J., Laxman, B., Mehra, R., Lonigro, R.J., Li, Y. *et al.* (2009) Metabolomic profiles delineate potential role for sarcosine in prostate cancer progression. *Nature*, **457**, 910-914.

30. Stabler, S., Koyama, T., Zhao, Z., Martinez-Ferrer, M., Allen, R.H., Luka, Z., Loukachevitch, L.V., Clark, P.E., Wagner, C. and Bhowmick, N.A. (2011) Serum methionine metabolites are risk factors for metastatic prostate cancer progression. *PLoS ONE*, **6**, e22486.

31. Angelucci, A., Garofalo, S., Speca, S., Bovadilla, A., Gravina, G.L., Muzi, P., Vicentini, C. and Bologna, M. (2008) Arachidonic acid modulates the crosstalk between prostate carcinoma and bone stromal cells. *Endocr Relat Cancer*, **15**, 91-100.

32. Thysell, E., Surowiec, I., Hornberg, E., Crnalic, S., Widmark, A., Johansson, A.I., Stattin, P., Bergh, A., Moritz, T., Antti, H. *et al.* (2010) Metabolomic characterization of human prostate cancer bone metastases reveals increased levels of cholesterol. *PLoS ONE*, **5**, e14175.

33. Attard, G., Belldegrun, A.S. and de Bono, J.S. (2005) Selective blockade of androgenic steroid synthesis by novel lyase inhibitors as a therapeutic strategy for treating metastatic prostate cancer. *BJU Int*, **96**, 1241-1246.

34. Nie, D., Che, M., Grignon, D., Tang, K. and Honn, K.V. (2001) Role of eicosanoids in prostate cancer progression. *Cancer Metastasis Rev*, **20**, 195-206.

35. Ferry, S., Matsuda, M., Yoshida, H. and Hirata, M. (2002) Inositol hexakisphosphate blocks tumor cell growth by activating apoptotic machinery as well as by inhibiting the Akt/NFkappaB-mediated cell survival pathway. *Carcinogenesis*, **23**, 2031-2041.

36. Gewinner, C., Wang, Z.C., Richardson, A., Teruya-Feldstein, J., Etemadmoghadam, D., Bowtell, D., Barretina, J., Lin, W.M., Rameh, L., Salmena, L. *et al.* (2009) Evidence that inositol polyphosphate 4-phosphatase type II is a tumor suppressor that inhibits PI3K signaling. *Cancer Cell*, **16**, 115-125.

37. Vucenik, I. and Shamsuddin, A.M. (2003) Cancer inhibition by inositol hexaphosphate (IP6) and inositol: from laboratory to clinic. *J Nutr*, **133**, 3778S-3784S.

38. Dann, S.G. and Thomas, G. (2006) The amino acid sensitive TOR pathway from yeast to mammals. *FEBS Lett*, **580**, 2821-2829.

39. Wang, X. and Proud, C.G. (2006) The mTOR pathway in the control of protein synthesis. *Physiology (Bethesda)*, **21**, 362-369.

40. Cook, S.J. and Morley, S.J. (2007) Nutrient-responsive mTOR signalling grows on Sterile ground. *Biochem J*, **403**, e1-3.

41. Wakshlag, J.J., Kallfelz, F.A., Wakshlag, R.R. and Davenport, G.M. (2006) The effects of branched-chain amino acids on canine neoplastic cell proliferation and death. *J Nutr*, **136**, 2007S-2010S.

42. Parker, W.B. (2009) Enzymology of purine and pyrimidine antimetabolites used in the treatment of cancer. *Chem Rev*, **109**, 2880-2893.

43. Schulze, A. and Harris, A.L. (2012) How cancer metabolism is tuned for proliferation and vulnerable to disruption. *Nature*, **491**, 364-373.
